# Methods used and lessons learnt in conducting document reviews of medical and allied health curricula – a key step in curriculum evaluation

**DOI:** 10.1186/1472-6920-14-236

**Published:** 2014-11-02

**Authors:** Anke Rohwer, Anel Schoonees, Taryn Young

**Affiliations:** Centre for Evidence-based Health Care, Faculty of Medicine and Health Sciences, Stellenbosch University, Francie van Zijl drive, 7500 Cape Town, South Africa; Community Health Division, Faculty of Medicine and Health Sciences, Stellenbosch University, Francie van Zijl drive, 7500 Cape Town, South Africa

**Keywords:** Curriculum review, Document review, Medical curriculum, Allied health curriculum, Evidence-based health care

## Abstract

**Background:**

This paper describes the process, our experience and the lessons learnt in doing document reviews of health science curricula. Since we could not find relevant literature to guide us on how to approach these reviews, we feel that sharing our experience would benefit researchers embarking on similar projects.

**Methods:**

We followed a rigorous, transparent, pre-specified approach that included the preparation of a protocol, a pre-piloted data extraction form and coding schedule. Data were extracted, analysed and synthesised. Quality checks were included at all stages of the process.

**Results:**

The main lessons we learnt related to time and project management, continuous quality assurance, selecting the software that meets the needs of the project, involving experts as needed and disseminating the findings to relevant stakeholders.

**Conclusion:**

A complete curriculum evaluation comprises, apart from a document review, interviews with students and lecturers to assess the learnt and taught curricula respectively. Rigorous methods must be used to ensure an objective assessment.

## Background

When tasked with conducting a document review of health science curricula to understand the current level of evidence-based health care (EBHC) teaching at Stellenbosch University (SU), we were unsure of the approach to such a study. Despite various attempts at searching electronic databases for published examples, we could not find relevant literature on the methodology of reviewing curriculum documentation and had to develop our own. We therefore feel that sharing the process of and our experiences in doing document reviews could assist other researchers when embarking on similar projects.

### Rationale for doing a document review

The importance of teaching EBHC to healthcare professionals has been highlighted in recent years [1–3]. As part of the SU Rural Medical Educational Partnership Initiative (SURMEPI), we, at the Centre for Evidence-based Health Care at SU in South Africa (http://www.cebhc.co.za), have been working on enhancing EBHC competencies of medical graduates since 2011. In order to enhance EBHC teaching, we needed to take stock of the extent to which EBHC competencies were already covered in the medical curriculum. This was in line with Kern’s first step in curriculum development, which entails identifying the problem and doing a general needs assessment by establishing the difference between the ideal and the current teaching approach. Kern describes that the current teaching approach is best assessed by reviewing the relevant documentation and interviewing medical educators, healthcare professionals, students and patients [4]. We started our situational analysis by reviewing the documentation of the undergraduate six-year medical curriculum.

About a year later, in 2013, we performed a similar exercise for the undergraduate allied health programmes at SU (Human Nutrition; Physiotherapy; and Speech, Language and Hearing Therapy). For this second document review, we drew on our previous experience and refined the methodology.

### Aim of this paper

This paper describes the process, our experience and the lessons learnt in doing document reviews of the undergraduate medical and allied health curricula at SU. The results of these studies are reported elsewhere.

## Methods

### Document review process

We followed a rigorous, systematic and transparent approach [5], which included writing protocols for both document reviews in which we pre-specified our intentions and included pre-piloted data extraction forms. The document review of the medical curriculum (N11/07/205) and of the allied health curricula (S12/10/261) were approved by the Stellenbosch University Ethics Committee of the Faculty of Medicine and Health Sciences.

Our process of doing a document review is explained in the following steps:

1. Defining competencies

**Figure 1:**
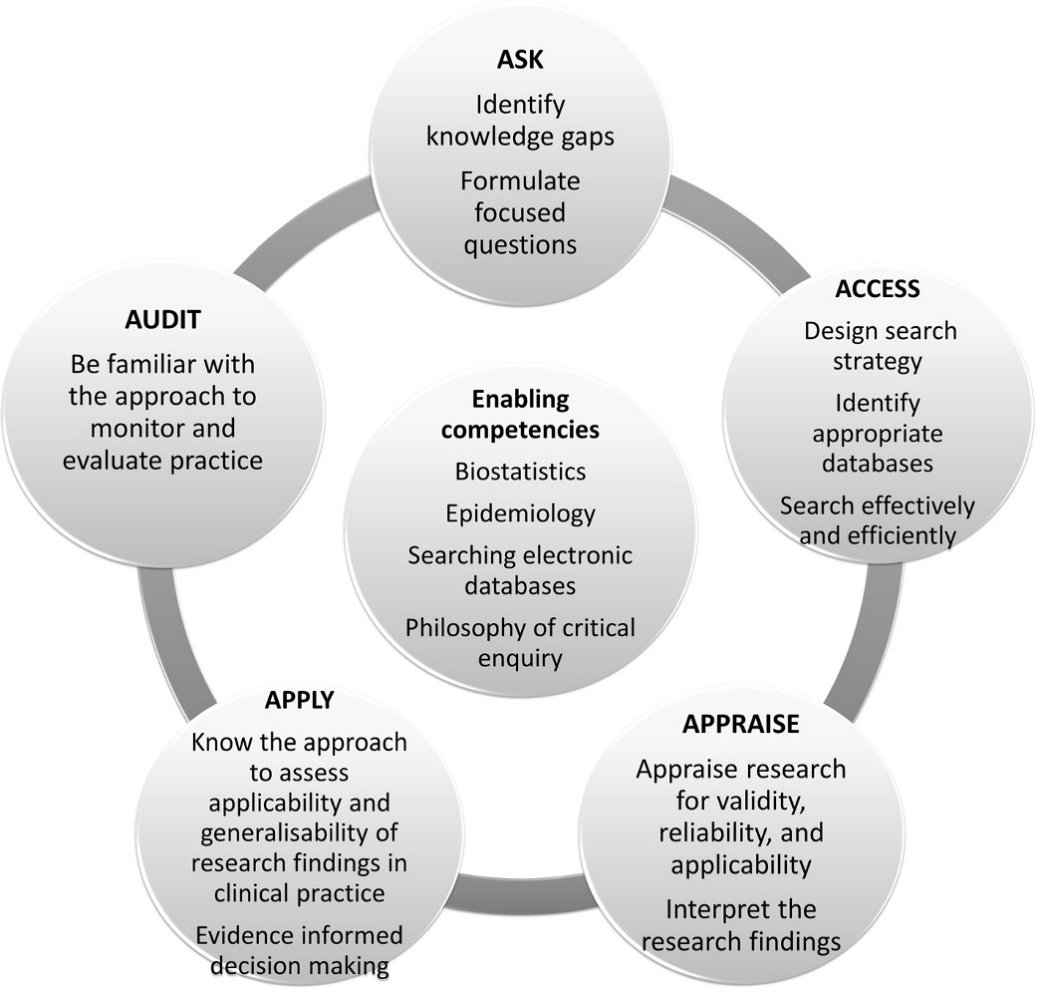
**Undergraduate enabling and key EBHC competencies.**

We started by doing a literature review on international studies regarding past and current trends in teaching and learning EBHC. We made use of a snowball technique, where we identified relevant studies from a search in PubMed and located additional studies by examining the reference lists. Drawing on this and on the CanMEDS framework [6] for graduate competencies, we developed a set of enabling and key EBHC competencies. Enabling competencies, which are a sub-set of competencies needed to achieve the key competencies, include the basic understanding of epidemiology and biostatistics, basic searching skills and the philosophy of critical enquiry. The five key competencies mirror the five steps of EBHC, namely i) formulating an answerable question based on clinical uncertainty; ii) finding the best available evidence to answer this question; iii) critically appraising and interpreting the evidence; iv) applying the results in the clinical setting; and v) evaluating the performance [3]. We discussed these proposed competencies with other faculty members and external experts before finalising them (Figure 1) [7].

2. Obtaining all module outlines and understanding the structure of the curriculum

We collected all relevant curriculum material and module outlines which detail module objectives, learning outcomes, and assessments. We collected 64 module outlines for the medical curriculum (2011) and 86 for three (Human Nutrition, Physiotherapy, Speech, Hearing and Language Therapy) of the four allied health curricula (2013). (At the time of our study, the Occupational Therapy division was revising their curriculum.) It was important to get a good understanding of how the curriculum phases flow and most importantly what is covered within which phase or year of study, whether modules are theoretically or clinically orientated, and which modules are presented to more than one programme to avoid duplicate data collection and extraction.

3. Extracting relevant data

To ensure consistency and standardisation in data extracted from the medical curriculum, we designed and piloted an electronic data extraction form in Microsoft Word. Learning outcomes, as documented in the module outlines (typically statements beginning with the following phrase *“After this session, students should be able to…”*), that related to the pre-specified EBHC competencies, were seen as the units of analysis. Within the team we discussed what information would be needed to obtain an accurate picture of the extent of and the approach to EBHC teaching and learning. We concluded that information on the data extraction form should include year and phase of study; name of the module; department or division responsible for teaching; specific learning opportunities and objectives/outcomes relevant to EBHC; method of teaching (face to face, tutorials, clinical rotations etc.); and assessment. We also aimed to record whether assessments were aligned to the learning outcomes, but assessments were poorly described (if at all) and we could not extract these data.

For the document review on the three allied health programmes, we developed and pre-piloted a set of codes based on the previously developed enabling and key EBHC competencies. What was different compared to the medical document review was that we did not extract data by entering it into a Microsoft Word table, but rather coded the relevant learning outcomes using ATLAS.ti software (ATLAS.ti Scientific Software Development GmbH). We had codes for each of the following categories: learning outcomes (with sub-codes for each pre-specified key and enabling competency), type of competency (sub-codes for knowledge (which included cognitive functioning), attitude or skill), mode of teaching (sub-codes for face-to-face, class exercises or tutorials, e-learning, self-study, or practical), and assessment (test and exam, assignment, practical assessment, web-based test, and other).

In both instances we performed quality checks on identified data. With the medical curriculum, a research assistant initially performed all data extraction. When one of the authors (AR) did quality checks on 30% of the data, she found very poor agreement (less than 30%) and thus re-extracted data from all raw documents. Uncertainties were resolved through discussions with another author (TY).

For the allied health document reviews, one team member identified all potentially relevant learning outcomes and coded each programme’s curriculum separately. One of the authors (AR) went through 30% of the module outlines and agreed that all potentially relevant outcomes had been identified and extracted. Two authors (AR, AS) then went through each extracted learning outcome, double-checked its relevance to EBHC as per the pre-specified competency list, and made the final decision regarding the choice of coding, i.e. the EBHC competency that it addressed.

4. Analysing data

Two authors (AR and either TY or AS) analysed the extracted data. For knowledge outcomes, we made judgements of the corresponding level of cognitive functioning by matching the verbs contained in the learning outcome to those used for each level of Bloom’s taxonomy. We used Table 1 [8] as a guide for judgement. When a learning outcome contained multiple verbs, we used the verb representing the highest cognitive level to make our judgement.

**Table 1:** **Bloom’s taxonomy and the associated verbs for each level of cognitive functioning**

Bloom’s level of cognitive functioning	Verbs describing the learning outcome
Knowledge	define, describe, identify, know, label, list, match, name, outline, recall, recognize, reproduce, select, state
Comprehension	comprehend, convert, defend, distinguish, estimate, explain, extend, generalize, give examples, infer, interpret, paraphrase, predict, rewrite, summarize, translate
Application	apply, change, compute, demonstrate, discover, manipulate, modify, operate, predict, prepare, produce, relate, show, solve, use
Analysis	analyse, break down, compare, contrast, diagram, deconstruct, differentiate, discriminate, distinguish, identify, illustrate, infer, outline, relate, select, separate
Synthesis	categorize, combine, compile, compose, create, devise, design, explain, generate, modify, organize, plan, rearrange, reconstruct, relate, reorganize, revise, rewrite, summarize, tell , write
Evaluation	appraise, compare, conclude, contrast, criticise, critique, defend, describe, discriminate, evaluate, explain, interpret, justify, relate, summarize, support

Examples illustrating how we classified learning outcomes according to knowledge, skill or attitude:

“Define epidemiology, disease incidence, prevalence, remission rates and death rates”.

“Explain the following terms: epidemiology, prevalence, incidence, endemic, epidemic, pandemic”.

Example 1: Learning outcome referring to knowledge:

We judged the first outcome as being relevant to EBHC and related it to the “knowledge” level of cognitive functioning because it contained the verb “define”. The second outcome, also relevant to EBHC, was related to the “comprehension” level, since it contained the verb “explain”.

Example 2: Learning outcome referring to attitude

Example 3: Learning outcome referring to skill

“The foundation has been laid for the ability to integrate and interpret knowledge and to think and act in a problem-solving way”.

“Formulate answerable background and foreground questions on diagnosis, therapy, prognosis and harm”.

5. Synthesising data

For the medical curriculum, we synthesised the data according to the three phases of study. Phase I includes all, except one, of the modules in the first year of study. Phase II includes one module from the first year and continues until the end of the first semester of the fifth year of study. It includes all theory modules, early and middle clinical rotations. Phase III, also called the student internship, includes the late clinical rotations and extends over the second semester of the fifth year and the sixth year of study. Learning outcomes that addressed the same EBHC competencies in a specific module were grouped together. The aim was to bring together elements related to teaching approaches, outcomes and assessment and relate this to the specific competencies. Table 2 is an example of the synthesised results of EBHC competencies in the first phase of the medical curriculum.

**Table 2:** **EBHC competencies in Phase I of the medical curriculum**

Competencies	Content covered	Module	Theme	Sessions	Teaching approach	Level of Bloom’s Taxonomy	Assessment methods
Enabling competency: Critical Thinking	Problem-solving	Personal and Professional development	4	Not applicable (N/A)	Lecture	N/A	Module guide states: continuous assessment, but no details are provided
Enabling competency: Biostatistics	Descriptive statistics	Health in context	2	5,6,7,9,14,15, 19	Lecture and discussion	Knowledge	Self-assessment and continuous assessment, but no details provided
				8,9,16	Practical	Application	
Enabling competency: Epidemiology	Study design	Health in context	2	1,2,3	Lecture	Knowledge	
	Measures of disease occurrence	Health in context	2	10,11,17,20	Lecture	Knowledge	
				10,11	Practical	Understanding	
		Essentials of disease processes	3	1,2	Lecture	Knowledge	Short test, class test and exam, but no details provided in the study guide
			13	1	Lecture	Understanding	
	Measures of association and effect	Health in context	2	12,13	Lecture	Knowledge	Self-assessment and continuous assessment, but no details provided
	Populations and sampling			18	Lecture	Understanding	
	Diagnostic accuracy			21,22,23	Lecture	Knowledge	
Key competency: Critical appraisal	Criteria/approach used to evaluate clinical study data	Principles of therapy	6	1,2	Lecture, tutorials	Understanding	Test, exam

The allied health programmes are not structured in phases like the medical curriculum, and the module outlines did not allow us to extract data regarding the teaching approach and assessment. We adapted the medical curriculum analysis table and constructed one table per programme, detailing competencies and content covered, year and module in which the competency is addressed and level of Bloom’s taxonomy. In addition, we added a quote of a typical learning outcome that addressed the respective competency. Table 3, displaying the key competencies of the Physiotherapy programme, is an example of how we presented results for the allied health curricula review.

**Table 3:** **EBHC key competencies within the undergraduate Physiotherapy (PT) programme**

Competencies	Content covered	Year	Module	Level of Bloom’s Taxonomy	Typical quotes
Principles of EBHC	Terminology and understanding	3,4	Research Methods 372, PT practice 74, Research Methods 472	Comprehension	Understand the principles of evidence- based practice
Formulating questions	Identify knowledge gaps	-	-	-	-
	Using PICO format	3	Research Methods 372	Application	Be able to design a secondary research question while using the PICO method
	Identify various types of questions		-	-	-
Literature search strategy	Identify best study design for a specific type of question		-	-	-
	Design a relevant search strategy		-	-	-
	Identify appropriate databases		-	-	-
	Performing an electronic search	3,4	Applied PT 373, Research Methods 372, Applied PT 473	Skill	Be able to search effectively for published PT research articles using the most common medical databases
Critical appraisal	Appraise systematic reviews		-	-	-
	Appraise randomised controlled trials		-	-	-
	Appraise cohort studies		-	-	-
	Appraise case-control studies		-	-	-
	Appraise cross-sectional studies		-	-	-
	Appraise diagnostic studies		-	-	-
	Appraise qualitative studies		-	-	-
	Interpret research findings		-	-	-
	Translate outcomes into summary statistics		-	-	-
	Appraisal not linked to specific study design	3,4	Applied PT 373, Applied PT 473, Clinical PT 374 ,Clinical PT 474, Research Methods 372, Research Methods 472	Application, Analysis	Be able to evaluate literature using PT-related critically appraisal tools
Applying the evidence	Consider application of relevant research		-	-	-

6. Compiling the report and disseminating findings

The final step involved compiling detailed reports, summaries and presentations of our findings and disseminating these to the relevant stakeholders – lecturers, heads of the various divisions and SURMEPI project partners. In addition, we presented the findings of our document reviews at various national and international conferences where a lot of interest was shown in the methodology we used.

## Results and discussion

### Lessons learnt and recommendations

While doing these document reviews, we experienced some challenges and stumbling blocks. Below are the most important lessons we learnt – some are generic while others are context specific.

- **Allow enough time:** Never underestimate how long it takes to do a rigorous document review, even though it might seem simple. Initially we thought it would not take longer than six months. However, for the allied health document reviews, it took five months to merely collect all module outlines. After realising that we needed to send numerous emails to obtain all relevant curriculum documents, we started data coding the module outlines that we had already received in an effort to save time. In the end, each document review took more than a year from conception until completion, taking into account that we wanted to ensure methodologically sound end-products.
- **On-going project management:** When working in a large team, it is essential that there is good communication and mutual understanding of what needs to be achieved. This includes scheduling regular meetings to keep everybody up-to-date and checking that everybody is working towards the same goal. Misunderstandings are inevitable; thus allowing time for sorting them out is important.
- **Continuous quality assurance:** We cannot overemphasise the importance of quality assurance at each stage of the study, especially when involving research assistants and consultants that are not part of the author team. This ensures that the process is rigorous and that no important information is lost.
- **Involve experts:** Acknowledge gaps in your knowledge and involve experts that can help fill these gaps. We invited an expert in medical education to join our team, since none of the members of the original working group had this expertise. For example, understanding Bloom’s taxonomy required repeated explanation and clarification before we agreed on how to apply it in our context. Table 1, which gave us explicit examples of how to categorise learning outcomes, was valuable as guidance.
- **Use software to organise data:** Select appropriate software to extract, analyse and manage data. For the medical document review, we completed electronic data extraction forms in Microsoft Word and ended up with over hundred files that needed to be organised. Using ATLAS.ti software for the subsequent document reviews made it much easier to identify, analyse and manage relevant data because it allows quick organising of the same codes. We used Dropbox to share files, enabling authors to have access to the latest versions of the working documents. This saved a lot of time and effort.
- **Disseminate findings:** Disseminate your findings to relevant stakeholders, even if they do not agree with your suggestions for improvement. Our results showed that EBHC teaching was mostly fragmented and that there was no progression from a lower to a higher level of cognitive functioning throughout the study programmes. When presenting the results to faculty members, we were mostly told that the learning outcomes did not reflect actual teaching in the classrooms or at the bedside.

## Conclusion

A document review of the formal curriculum on its own might not reflect the true teaching and learning experienced by the students, but provides a valuable point of departure for further investigations. It is thus an important baseline study that needs to be conducted with rigor and precision. We emphasise that a document review should be part of a three-pronged approach, including information gained from interviews with lecturers (taught curriculum) and students (learned curriculum).

## Abbreviations

EBHC: Evidence-based Health Care
SU: Stellenbosch University
SURMEPI: Stellenbosch University Rural Medical Education Partnership Initiative.
